# Mobile Health Features Supporting Self-Management Behavior in Patients With Chronic Arthritis: Mixed-Methods Approach on Patient Preferences

**DOI:** 10.2196/12535

**Published:** 2019-03-25

**Authors:** Jonas Geuens, Luc Geurts, Thijs W Swinnen, Rene Westhovens, Vero Vanden Abeele

**Affiliations:** 1 e-Media Research Lab Katholieke Universiteit Leuven Leuven Belgium; 2 Division of Rheumatology Universitaire Ziekenhuizen Gasthuisberg University Hospitals Leuven Leuven Belgium; 3 Department of Development and Regeneration Skeletal Biology and Engineering Research Center Katholieke Universiteit Leuven Leuven Belgium

**Keywords:** mobile applications, arthritis, self-management

## Abstract

**Background:**

Patients with chronic arthritis (CA) ideally apply self-management behaviors between consultations. This enduring, tedious task of keeping track of disease-related parameters, adhering to medication schemes, and engaging in physical therapy may be supported by using a mobile health (mHealth) app. However, further research is needed to determine which self-management features are valued most by adult patients with CA patients.

**Objective:**

The aim of this study was to determine the preference of features for an mHealth app to support self-management behavior in patients with CA. In addition, we aimed to explore the motives behind these ratings.

**Methods:**

A mixed-methods approach was used to gather information from 31 adult patients (14 females), aged 23 to 71 years (mean 51 [SD 12.16]), with CA. Structured interviews were conducted to gather data pertaining to preferences of app features. Interviews were analyzed qualitatively, whereas ratings for each of the 28 features studied were analyzed quantitatively.

**Results:**

In general, patients with CA favored the use of features pertaining to supporting active and direct disease management, (eg, medication intake and detecting and alarming of bad posture), helping them to keep a close watch on their disease status and inform their health care professional (eg, providing a means to log and report disease-related data) and receiving personalized information (eg, offering tailored information based on the patient’s health data). Patients strongly disliked features that provide a means of social interaction or provide incentivization for disease-related actions (eg, being able to compare yourself with other patients, cooperating toward a common goal, and receiving encouragement from friends and/or family). Driving these evaluations is the finding that *every patient with CA hurts in his/her own way*, the way the disease unfolds over time and manifests itself in the patient and social environment is different for every patient, and patients with CA are well aware of this.

**Conclusions:**

We have offered an insight into how patients with CA favor mHealth features for self-management apps. The results of this research can inform the design and development of prospective self-management apps for patients with CA.

## Introduction

### Background

Chronic arthritis (CA) is an umbrella term for inflammatory diseases that affect about 20% of the US and European population [1,2]. Symptoms include joint pain, swelling, stiffness, and instability and joint destruction or bony ankylosis, resulting in progressive impairment of mobility when insufficiently controlled [3]. Patients are often limited in their day-to-day activities owing to these symptoms. Effective drugs to treat CA are available but require a long-term commitment. In addition, patients are recommended to participate in frequent physical therapy to improve mobility, cardiovascular endurance, postural control, and muscle strength. Increasingly, self-management is becoming a basic principle in treatment efforts. Patients are required to keep a close watch on their disease parameters to swiftly identify changes in disease status and adapt medication intake or exercise regimens. Thus, managing CA is a complex and demanding activity [2]. Not surprisingly, patients often fail to comply with this strict and enduring treatment regimen [4-6]. This is unfortunate, as long-term health outcomes depend on these self-management behaviors and as successful self-management of CA promotes physical and emotional well-being of the patient and reduces health care costs [7,8].

### Supporting Self-Management Behaviors

A possible (partial) solution may come in the shape of mobile health (mHealth) apps incorporating features to support and motivate patients with CA to engage in and adhere to self-management behaviors. Several theories and models exist to inform mHealth app designers in which such features may motivate and support patients. The Persuasive System Design (PSD) model [9] starts from the assumption that technology can be designed to change attitudes or behaviors through persuasion and social influence [10]. Therefore, the PSD model contains 28 design principles divided over 4 umbrella categories: primary task support (eg, setting tailored goals), dialogue support (eg, sending reminders), social support (eg, providing social norms), and system credibility support (eg, listing third-party endorsements). Another established framework, the taxonomy of Behavior Change Techniques (BCTs) incorporates 26 techniques to inspire mHealth designers [11]. Being different from the PSD model, BCT principles do not start from *persuasive design principles* but rather emanate from *behavior change theories*. Consequently, authors link their techniques to the underlying theories of behavior change, for example, the technique of giving rewards is linked to operant conditioning.

Several researchers use one or a combination of the aforementioned frameworks, for example, to evaluate the presence of features to support self-management behaviors [9,12-18]. Despite different underlying epistemologies, several PSD principles and BCTs overlap in the way they manifest themselves in mHealth apps. Therefore, Geuens et al [19] compiled the 2 frameworks and presented a list of 28 unique *motivational* features to support and motivate self-management behaviors in mHealth apps.

### Presence of and Preference for Self-Management Features in Mobile Health

With regard to CA, Geuens et al [20] conducted a systematic review of persuasive principles and BCTs present in current health apps. The authors coded 28 mHealth apps. They found that the most used category of persuasive principles was system credibility, in particular, avoiding banners and advertisements and providing information on who contributed to the development of the app. Task support was the second most used category and mainly comprised the option to compute a Disease Activity Score. Only a few apps supported physical exercise. Next was dialogue support, consisting of sending out reminders with respect to medication intake. Surprisingly, social support principles were lacking in all but one app.

Although the aforementioned study provides information on what self-management features are found in mHealth apps, it does not inform us of how these features are evaluated by patients with CA *themselves*. In 2015, Revenäs et al conducted 4 consequent workshops with 5 adult patients with rheumatoid arthritis, 5 health care professionals, and 2 Web developers [21]. They found that patients with CA preferred 2 major components for a Web-based or mobile app: a calendar for goal setting, planning, and recording of disease-related parameters and a community to receive support from peers. Another study by Revenäs et al with 26 individuals with rheumatoid arthritis, not on mHealth but internet services, also identified several key features to support physical activity for patients with rheumatoid arthritis [22]. They identified the following core features: up-to-date and evidence-based information, self-regulation tools, social interaction, personalized set-up, attractive design, and access to the internet service. To the best of the author’s knowledge, no other studies report on preferred self-management mHealth features with *adult patients with CA.* This is unfortunate as a lack of patient involvement in the design and selection of self-management features in apps may negatively impact acceptance of such mHealth apps.

However, recent studies document the preferences of patients with juvenile idiopathic arthritis (JIA), aged 10 to 24 years. Waite-Jones et al [23] conducted semistructured focus groups and individual interviews with 9 young people, 8 parents, and 8 health care professionals. The findings from this study suggested that an app for self-management of juvenile arthritis should provide young people with the ownership and control of an engaging tool that (1) gives information, (2) monitors symptoms, (3) offers reminders, and (4) provides social support. Cai et al [24] equally used focus groups as part of a qualitative, user-centered design approach involving 29 young people with JIA, 7 parents, and 21 health care professionals from the rheumatology team. The major themes that they identified to inform app development were (1) remote monitoring of symptoms such as pain and swelling/stiffness of joints, overall mood, stress and sleep, and physical activities, well-being; (2) treatment adherence, that is, tracking medication and exercise schemas and sending out reminders; (3) education and support (giving links to educational sites, support groups and JIA-related services, and providing information on juvenile arthritis); and, in later phases, themes related to the following were also mentioned: (4) providing incentives, (5) privacy, (6) ease-of-use, (7) integration of clinical tools support, and overall (8) attractiveness of design.

These qualitative studies on JIA inform us of desired design features for mHealth apps by young patients with JIA and their caretakers. However, mHealth features that are found supportive by older patients with CA are only studied in the aforementioned work by Revenäs et al. Apart from the medical differences [25], patients with juvenile and adult arthritis may have age-related differences in disease self-management. When using mHealth for self-management of CA, differences in technology proficiency between adolescents and adults may become apparent [26], resulting in a different way of using mHealth apps. Wyatt et al [27] recommended age specificity as one of the key elements for a good design of an mHealth app. Hence, there is a clear need to further study preferences of adult patients with CA with regard to mHealth features supporting disease management.

### The Contribution of This Study

In this study, we aimed to evaluate, in a quantitative and qualitative manner, how adult patients with CA evaluate design features supporting self-management behaviors embedded in mHealth apps. First, we aimed to understand *which features are rated positively and which are rated negatively*. Second, we aimed to explore the reasons patients with CA provide to explain these scorings, in a qualitative manner. *What are the reasons underlying positive or negative scorings?* The broader goal of this study was to inform researchers and developers of mHealth apps on which features are desired by patients with CA themselves, to promote long-term adoption of the app.

## Methods

This study employed a mixed-methods approach, a quantitative analysis of how patients rated mHealth features and a qualitative analysis of interviews.

### Participants

Participants (see Table 1) were randomly selected from patients visiting the medical ambulatory center of the rheumatology division of the University Hospital Gasthuisberg in Leuven, Belgium, in the fall of 2017. Ethical approval for this study was granted by the ethics committee of the University Hospital Gasthuisberg with protocol number S-59012. Patients were recruited before their appointment with their rheumatologist. Information was provided about the intent of the interview and patients were asked to sign a consent form detailing the collection, processing, and storing of data collected during the interview. Patients could stop the interview at any time. Inclusion criteria were that patients should be diagnosed with CA and be at least 18 years. Data related to the current disease status (age, sex, years since diagnosis and disease-related parameters) were collected from the hospital’s registries, coupled with the respective patient’s interview data, and anonymized. We aimed for a purposively heterogeneous yet representative sample of patients with CA. In total, 31 patients (14 females), aged 23 to 71 years (mean 51 [SD 12.16]), were recruited over the course of 4 months. Patients with CA in this study varied with regard to their medical disease status.

### Data Collection and Analysis

Both qualitative and quantitative data were collected during the interviews. The qualitative data contained the complete recording of the interview, that is, answers to the first part of the interview (open questions related to disease management) and the second part (scorings on 28 features and additional comments). Audio recordings were made during the interviews and transcribed verbatim. Semistructured interviews were conducted before or after a consultation with a rheumatologist and took place in the same building. The first part of the interview started with an open question asking patients to describe how they currently managed their condition. Next, the interviewer followed up by asking about medication, physical therapy, and the use of technology to support them. The second part of the interview consisted of questions that polled the favorability of 28 possible features of an mHealth app for patients with CA (based on [19], see Textbox 1) supporting self-management behaviors. Patients with CA were asked to provide a Likert score between 1 (strong dislike) and 5 (strong like). In addition, patients were invited to further comment on why they gave this scoring and were encouraged to ask for clarification whenever the intent of a feature was not clear. After the 28 features were reviewed, the interviewer asked once more for additional comments on their evaluation. Interviews ranged between 8 and 31 min, with an average of 14 min.

### Qualitative Data Analysis

The qualitative data were entered and coded in NVivo 11 (QSR) according to a thematic analysis process as described by Braun and Clarke [32]. In the first phase, 2 researchers and coauthors of this paper (JG and VVA) familiarized themselves with the data and provided initial codes for all data (the data provided in the first part of the interview, the explanation of the rating for the 28 mHealth features, and the closing of the interview). In a next round, themes were derived, grouping the different mHealth features based on emerging topics. A final coding round was conducted, unearthing the underlying core concept and design implications (see Table 2).

### Quantitative Data

The scoring of patients with CA was based on the 5-point Likert scale, related to how patients evaluated the 28 features of an mHealth app, and was entered in Microsoft Excel for descriptive statistics. Given the face-to-face interview, there were no missing data. Data were further inspected according to the process described by Gaskin [30]. As a suspicious answer pattern was found (ie, limited variance and providing only ratings of 5), patient 19 was omitted from the dataset. SPSS was used for conducting 2 one-sample *t* tests (one-tailed, alpha=.05), with a Bonferroni correction (based on 28 tests) to correct for inflation of the false-positive error rate. Data were exported to comma-separated value files and imported in a Python [31] worksheet for further processing and the rendering of violin plots to illustrate sample distribution as well as a box plot.

**Table 1:** Patients participating in interviews and focus groups, with gender, age, and disease-related scores. Scores varied from 0.00 to 7.20 (out of a possible 0 to 10) on the Bath Ankylosing Spondylitis Disease Activity (BASDAI) (mean 4.02 [SD 2.26]) and 0.00 to 9.50 (out of a possible 0 to 10) on the Bath Ankylosing Spondylitis Functional Index (BASFI) (mean 4.07 [SD 2.66]).

Participant	Sex	Age (years)	Diagnosis (years)	BASDAI^a^	BASFI^b^
Patient 1	M	48	33	2.60	2.50
Patient 2	F	48	30	7.80	6.90
Patient 3	M	54	26	3.30	5.70
Patient 4	M	59	9	0.80	0.50
Patient 5	F	34	9	—^c^	—
Patient 6	M	55	39	1.80	5.70
Patient 7	M	66	47	3.40	4.10
Patient 8	F	71	46	4.40	5.00
Patient 9	M	47	22	6.40	9.50
Patient 10	F	51	18	3.60	2.90
Patient 11	M	45	10	3.50	5.20
Patient 12	M	23	10	0.80	0.00
Patient 13	F	58	27	7.00	6.70
Patient 14	M	63	44	0.00	0.00
Patient 15	F	41	12	7.10	8.10
Patient 16	F	59	14	6.50	7.30
Patient 17	F	56	7	4.00	2.20
Patient 18	F	69	34	2.70	1.90
Patient 19^d^	M	38	1	Excluded	Excluded
Patient 20	M	39	15	1.10	1.40
Patient 21	F	38	14	3.70	2.30
Patient 22	M	57	14	5.80	5.70
Patient 23	F	41	16	5.40	4.40
Patient 24	F	61	9	7.00	5.90
Patient 25	F	46	14	5.10	5.30
Patient 26	M	29	8	1.30	0.00
Patient 27	M	59	41	4.70	2.20
Patient 28	M	54	27	7.20	6.40
Patient 29	M	49	27	3.80	7.00
Patient 30	F	67	14	6.90	6.90
Patient 31	M	48	27	1.20	1.90
Average (%)	53 M	53	21	4.02	4.07
SD	—	12.16	12.65	2.26	2.66

^a^BASDAI: Bath Ankylosing Spondylitis Disease Activity [28].

^b^BASFI: Bath Ankylosing Spondylitis Functional Index [29].

^c^Missing data.

^d^Patient 19 was excluded from the quantitative analysis because he rated all features a 5.

**Mobile health (mHealth) features selection for which a 1 to 5 Likert rating was asked during the interview. Patients were asked for their rating of the feature which was asked for as described below. The general descriptor (marked in italics) is provided for the convenience of the reader but was not provided to the patient.**

*Disease activity scoring:* You are asked to enter measurement results in an app. The app automatically calculates several useful disease-related scores instead of you having to calculate these scores by hand.

*Exercise scheduling:* You want to be able to walk a distance of 5 miles in a few months. The app calculates the right exercise schedule to guide you toward this goal.

*Exercise instructions:* You are required to perform a set of exercises. The app provides detailed instructions on how to perform each exercise.

*Exercise assessment:* Sensors measure whether you are executing an exercise the right way. You are able to perform the exercise a few times before the measurement is actually started.

*Medication reminders:* You are required to take your medication at fixed intervals. The app reminds you when you need to take your medication.

*General Information:* You are able to read general information about arthritis in the app.

*Tailored information:* The information in the app is specific for your type of arthritis.

*Personalized information:* The information in the app is specific to you personally.

*Pain analysis:* The app is able to predict possible causes of pain from the collected data.

*Logs for reporting:* You are able to save information about your condition in the app to show to your physician.

*Disease tracking:* The app automatically collects data relevant to your disease.

*Graphs:* You are able to consult graphs and data based on your own data.

*Rewards:* You are able to collect rewards based on your execution of exercises.

*Praise:* You are encouraged during your physical therapy through motivational messages.

*Gamification:* Would you like the app to provide exercise instructions in a playful manner, for example, by using playful sounds or collecting badges or points?

*Social media sharing:* The app shows other users that you have been taking the most steps this week and you are able to share this on social media.

*Social identification:* You are able to view limited data of other users with the same condition.

*Social comparison:* You are able to compare yourself to other users

*Competition:* You are able to challenge other users to, for example, walk the longest distance.

*Cooperation:* You are able to work together with other users to achieve a common goal.

*Encouragement:* As you are executing your exercises, family and friends are able to send you motivational messages.

*Goal setting:* You are able to choose your goal, and the app will guide you toward this goal.

*Context-awareness:* The app tells you the weather is nice and there is a beautiful park nearby and suggests you go for a walk.

*Styling:* You are able to personalize the app, for example, change colors, set a profile picture, and choose what is shown.

*Posture detection:* The app detects bad posture and suggests correcting your posture.

*Verifiability:* The app shows scientific articles that describe the design and development of the app.

*Expertise:* The app shows physicians, therapists, and researchers that helped create the app.

*Surface credibility:* The app does not contain advertisements.

**Table 2:** Example of coding of the qualitative data into coding nodes, a category and subcategory.

Phase of the interview and content		mHealth^a^ feature	Grouping themes	Design implications	Core concept
**Start**					
	*Interviewer: To start the interview, can you tell how you manage the disease?*	—^b^	—	—	All patients hurt in their own way
	Patient: Well, you are confronted with it on a daily basis, it is actually a part of your life.	—	—	—	All patients hurt in their own way
**Rating and explanation of 28 mHealth features**					
	*Interviewer: While you are executing your exercises, family and friends are able to send you motivational messages through the app.*	Encouragement	Social interaction	No need for social sharing or comparing, CA^c^ is a private matter	All patients hurt in their own way
	Patient: For me, personally, that is not that important. I like to keep that private. My disease should not take the upper hand in my social encounters. I score it a 2.	—	—	—	All patients hurt in their own way
**Closing of the interview**					
	*Interviewer: Would you like to have an app containing these features?*	Posture detection	Disease action support	—	All patients hurt in their own way
	Patient: I think so. Perhaps not all features, I would use particularly the feature with posture.	—	—	—	All patients hurt in their own way
	*Interviewer: What [features] would you not use?*	—	Social interaction	No need for social sharing or comparing, CA is a private matter	All patients hurt in their own way
	Patient: Everything related to other users. All of that. The disease is very personal, it is different for everyone?	—	—	—	All patients hurt in their own way

^a^mHealth: mobile health.

^b^Not applicable.

^c^CA: chronic arthritis.

## Results

### General Results

**Figure 1:**
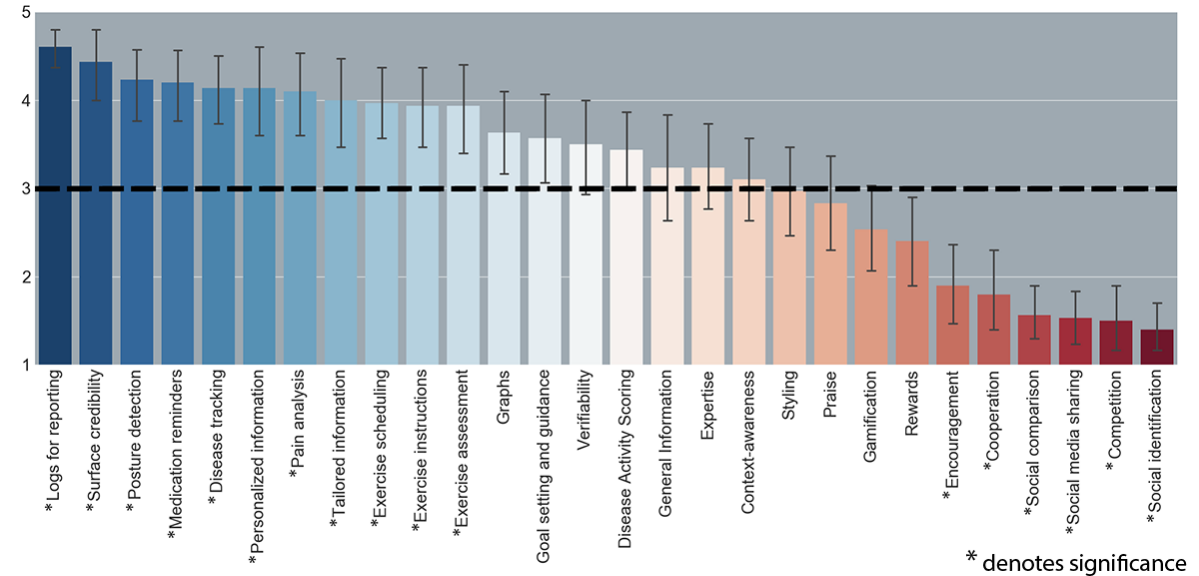
Scoring of features for a mHealth application for CA patients ( *denotes significance).

Overall, out of the 28 features supporting self-management behavior, 11 received a scoring significantly higher than 3 (*neither like* and *neither dislike*) and 6 received a scoring significantly lower than 3. Table 3 lists the features sorted on mean scorings with *t* values, *P* values, and CIs. Figure 1 provides an overview of the scorings, ordered from the highest average (left) to the lowest.

Upon qualitative analysis, we grouped features into the following themes to structure our results: *Disease action support*, *Disease insight*, *Information*, *Incentivization*, *Social interaction*, *Credibility*, and *Personalization*. Both quantitative and qualitative data will be discussed further below according to these themes. Next, we will argue for a core concept underlying these themes and scorings and provide implications for the selection and design of supportive self-management features.

### Themes and Mobile Health Features

#### Disease Action Support

**Figure 2:**
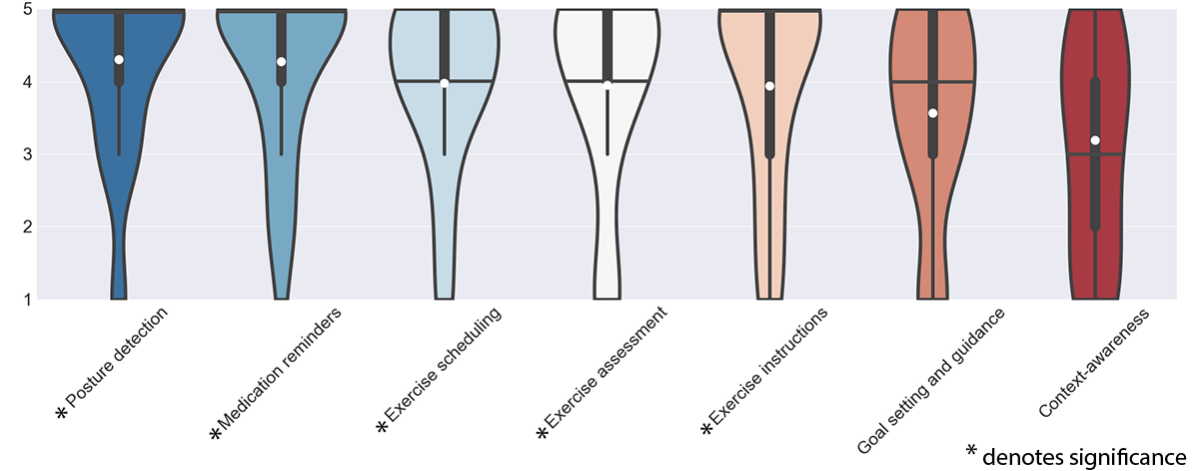
Scores of features in the group of disease action support. The white dot denotes the mean value, the horizontal black bar denotes the median value, the thick black bar denotes the interquartile range and the thin black bar denotes the 95% confidence interval.

The *Disease action support* theme contains those features that support patients’ *active* behaviors; executing disease related actions such as physical therapy, improving physical well-being, or medication intake all have a direct effect on health-related outcomes (Figure 2). In general, we found that patients with CA welcomed features that support the active management of their disease, all features scored above 3, of which 5 out of 7 were significant.

**Table 3:** Quantitative results of the interviews. Features are sorted based on the mean score.

Feature	Mean	*T* value	*P* value
Logs for reporting	4.60	14.102	<.001^a^
Surface credibility	4.43	6.916	<.001^a^
Posture detection	4.23	5.798	<.001^a^
Medication reminders	4.20	5.541	<.001^a^
Disease tracking	4.13	5.461	<.001^a^
Personalized information	4.13	4.41	<.001^a^
Pain analysis	4.10	4.748	<.001^a^
Tailored information	4.00	3.808	.001^a^
Exercise scheduling	3.97	4.455	<.001^a^
Exercise instructions	3.93	3.619	.001^a^
Exercise assessment	3.93	3.683	.001^a^
Graphs	3.63	2.567	.02
Goal setting and guidance	3.57	2.246	.03
Verifiability	3.50	1.822	.08
Disease activity scoring	3.43	1.692	.10
General information	3.23	0.763	.45
Expertise	3.23	0.879	.39
Context-awareness	3.10	0.379	.71
Styling	2.97	−0.126	.90
Praise	2.83	−0.604	.55
Gamification	2.53	−1.848	.08
Rewards	2.40	−2.34	.03
Encouragement	1.90	−4.557	<.001^b^
Cooperation	1.80	−5.288	<.001^b^
Social comparison	1.57	−8.746	<.001^b^
Social media sharing	1.53	−9.337	<.001^b^
Competition	1.50	−7.883	<.001^b^
Social identification	1.40	−11.379	<.001^b^

^a^Significant higher score than mean.

^b^Significant lower score than mean.

*Medication reminders* (μ_x_=4.20, σ_x_=1.17) was rated highest in this theme. Patients favored reminders on when to take medication:

That is top, the morning medication is not a problem, but the evening medication I sometimes forget because of other activities, so a 5.

Patient 18

Now, I do not experience problems with this as I have to take medication daily. But there have been periods where I had to take medication only twice a week, and that was more complicated. So yes, a 4.

Patient 21

The few patients who were less positive typically expressed they had other means to remind them:

I usually use my alarm clock to remind me about my medication, so let’s give this a 3 out of 5.

Patient 26

*Posture detection* (μ_x_=4.23, σ_x_=1.15) also scored highly positive. Patients liked an app to detect their posture and flag them when their posture was bad, as emphasized during the interviews by several patients:

[..] to use a sensor on their back to detect posture, and flagging whether it was good or bad. I do think this would be good for me to have. So, this is a feature to which I say yes.

Patient 23

Ideally, this would be a kind of clothing you can where and in which your posture measured at multiple points, and then tells you “You are sitting wrongly.”

Patient 3

*Exercise scheduling* (μ_x_=3.97, σ_x_=1.17), *Exercise instructions* (μ_x_=3.93, σ_x_=1.39), and *Exercise assessment* (μ_x_=3.93, σ_x_=1.36) received similar positive scores. Patients liked features to make them more physically active, provide instructions on how to do it, and particularly, how to do it well:

All initiatives [to get me moving more] are welcome, I can’t say no to that.

Patient 22

I find it particularly important that an app can give instructions on how to do it better. Other features I care less for.

Patient 26

You may exercise as often as you want, but if you do them wrong, there is absolutely no point. So, I definitely welcome anything that would help in this respect.

Patient 15

*Goal setting* (μ_x_=3.57, σ_x_=1.36) and *Context-awareness exercise support* (μ_x_=3.10, σ_x_=1.42) although still being rated above 3 did not reach significance. When asked whether they would want to be able to choose a goal and be guided toward this, some patients expressed skepticism about the capabilities of an mHealth app to be able to deliver. Patients also questioned whether the extent of personalization that is needed would be offered by an app:

This may be useful if it is personalized. You have to be able to make changes or signal when it is going too fast. Because this is really different for every patient. A healthy person can build endurance but someone who has pain, like me − I feel better one day compared to another − has to be able to get a pass from time to time. So, a 3 if I can make changes and indicate why and flag where it hurts.

Patient 3

As for context-awareness support, the same skepticism was encountered. Moreover, it seemed that some patients had a harder time imagining this.

Many patients also expressed their confidence in physical therapists over an mHealth app in this regard. In general, patients highly valued their sessions with the therapist and would not like to see them replaced by an app:

I think you’re better off going to a physical therapist where everything is explained in more detail and where you are shown how to do the exercises. They can also correct you when you’re doing an exercise the wrong way.

Patient 27

#### Disease Insight

**Figure 3:**
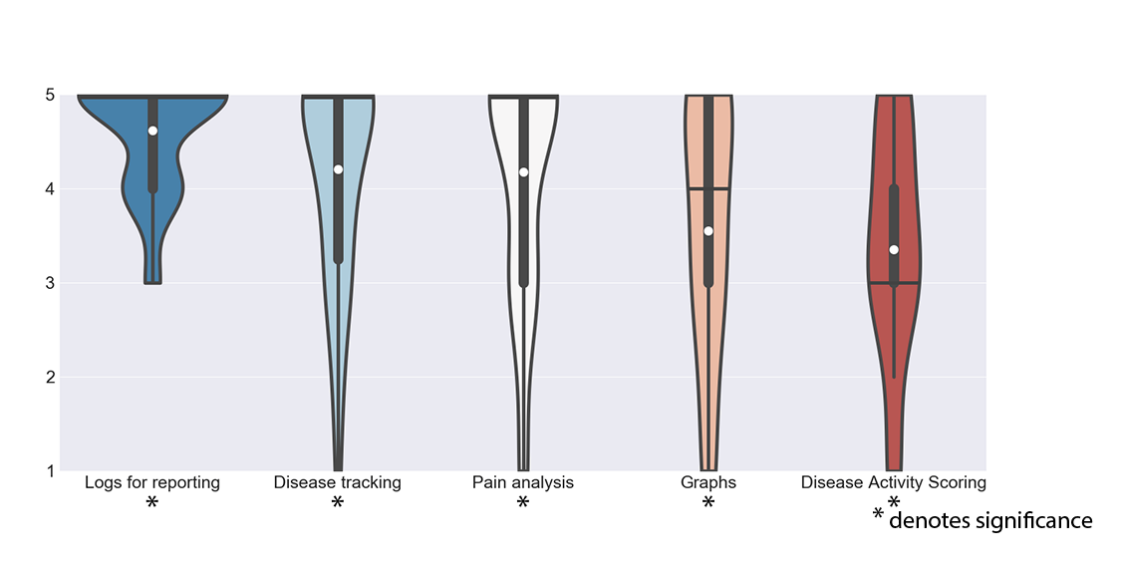
Scores of features in the group of disease insight. The white dot denotes the mean value, the horizontal black bar denotes the median value, the thick black bar denotes the interquartile range and the thin black bar denotes the 95% confidence interval.

The *Disease insight* theme contains features that allow patients to keep a closer watch on their disease and give a deeper insight and/or to communicate about their disease status to health professionals. However, contrary to the *Disease action support* theme, these features do not directly support actions, but rather, they may have an indirect impact on health-related outcomes (Figure 3). The features of this theme were also rated favorably by patients; all features were scored above 3, and 4 out of 5 features were significant.

*Logs for reporting* (μ_x_=4.60, σ_x_=0.61) received the highest rating of all 28 features. The interviewed patients favored the ability to log and save data related to their condition in the app and, in particular, to have a way to generate a report of these data to show to their rheumatologist:

That might be a good feature to have. I have an appointment with my rheumatologist every 8 weeks and every time I get home, I remember things I should have asked or mentioned.

Patient 30

It seems a useful feature because you forget things rapidly. When [the healthcare professionals] ask you questions but you have already forgotten that something has happened.

Patient 29

However, for this feature to be useful, patients also emphasized that it should be easy to use:

I’m on the edge. On one side, I think it’s great but on the other side… it has to be really easy to enter data, it should not take up too much time.

Patient 23

*Disease tracking* (μ_x_=4.13, σ_x_=1.12) and *Pain analysis* (μ_x_=4.10, σ_x_=1.25) were also highly favored. Patients liked the app to automatically collect data related to their condition and liked the app to predict the possible causes of pain based on collected data:

If that were possible, yes, please, a 5!

Patient 9

That seems interesting. You typically feel [the cause of pain] yourself but it couldn’t hurt to have a confirmation.

Patient 22

Yet, here too, patients were skeptical about an app being able to do this:

If it measures correctly because I currently have an app but that’s not impressive [...] It really has to be able to display reality.

Patient 23

*Graphs* (μ_x_=3.63, σ_x_=1.33) were rated positively by some but not all patients. Those patients who were in favor often linked the use of graphs to be able to communicate to professionals, similar to the afore-discussed *logs for reporting*:

I think [this feature] is really useful. If they ask me how my last week was and I feel bad right now, then my entire week was bad. If I had a graph, I could show them [my healthcare professionals].

Patient 3

*Disease activity scoring* (μ_x_=3.43, σ_x_=1.20) was rated lowest in this theme and did not reach significance. Although not expressing dislike, none of the patients currently did this, and consequently, most patients did not see the use for themselves:

I would rate this a 3 out of 5 because I wouldn’t use [the feature]. I think it’s only useful when your disease is still changing. My condition has been stable for the past 20−25 years.

Patient 27

I’m always asking myself: what’s the benefit for me? I’m not going to fill in another questionnaire when, in the end, I don’t know more than I know right now. My situation is optimal right now, I don’t have a lot of problems with my condition, I try not to think about my condition.

Patient 4

#### Information

**Figure 4:**
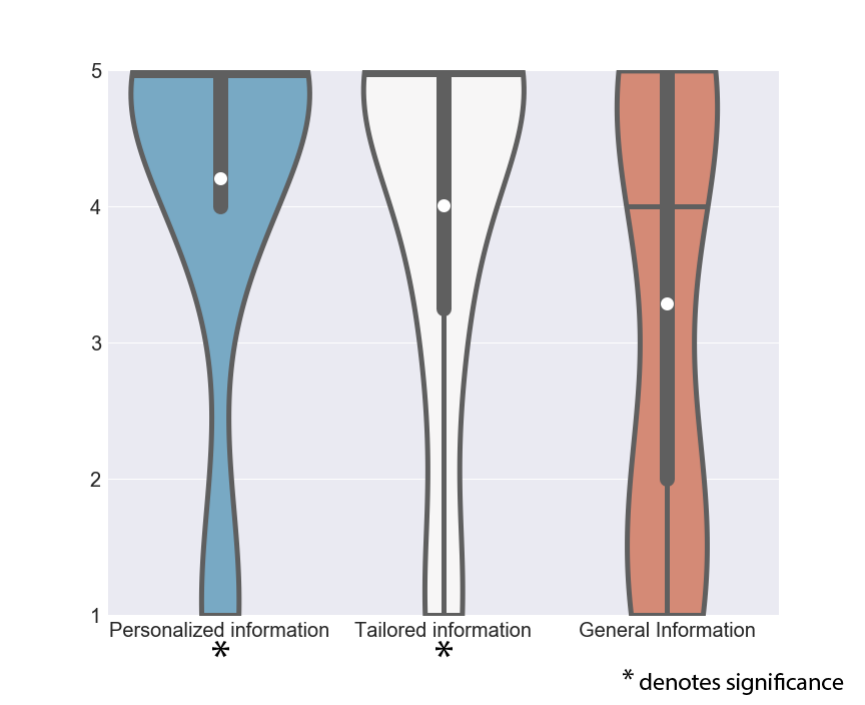
Scores of features in the group of Information. The white dot denotes the mean value, the horizontal black bar denotes the median value, the thick black bar denotes the interquartile range and the thin black bar denotes the 95% confidence interval.

The group of information contains those features that provide either general, tailored (disease-related, at the group level), or personal information on arthritis to the patient (Figure 4). Here scores vary depending on the degree of how tailored information is offered.

*Personalized information* (μ_x_=4.13, σ_x_=1.38) as well as *tailored information* (μ_x_=4.00, σ_x_=1.41) were found favorable by most patients, acknowledging a need for information to help them understand the disease better:

That seems useful. When you feel something you haven’t felt before […] You attribute every new pain you feel to your condition but maybe you are missing some valuable sign which has nothing to do with your disease.

Patient 3

*General information* (μ_x_=3.23, σ_x_=1.65), however, was rated only 3.23 out of 5 on average (SD 1.65). Patients did not feel a strong need to read general information related to arthritis in the app. In this case, they would use Google or ask their therapist:

You don’t need to create an entire book in an app. I can also receive information from my therapist.

Patient 27

I don’t need this. I can also Google everything.

Patient 3

#### Credibility and Styling

**Figure 5:**
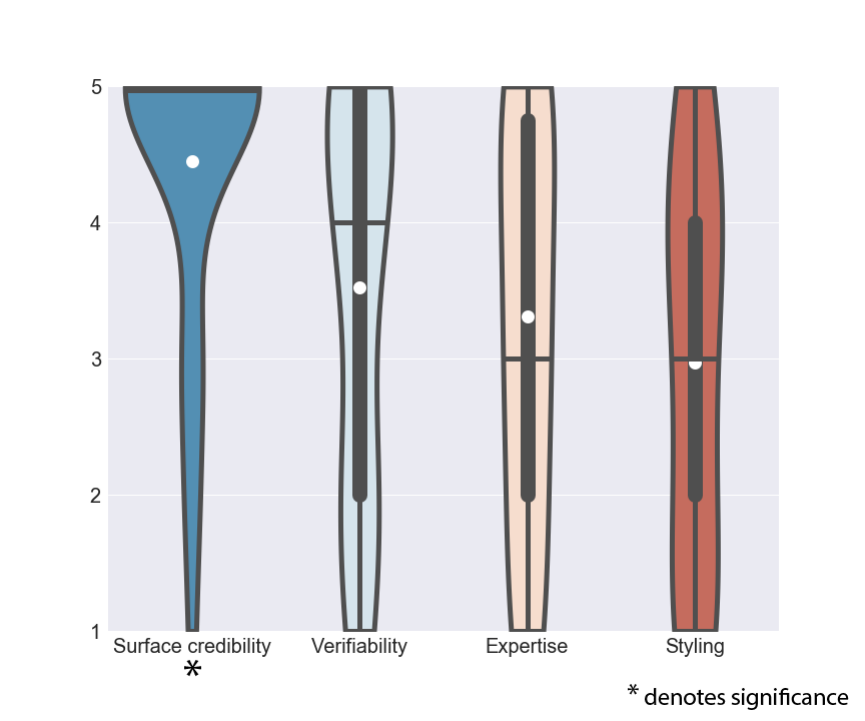
Scores of features in the group of Credibility & styling. The white dot denotes the mean value, the horizontal black bar denotes the median value, the thick black bar denotes the interquartile range and the thin black bar denotes the 95% confidence interval.

The group of credibility contains features related to how the app can increase perceived credibility or be styled to personal liking (Figure 5) by banning advertisements, providing information on the makers, or allowing styling. Scores diverge in this last group, whereas surface credibility (banning advertisements) was highly valued; other features are less outspoken, but still rated positively overall.

*Surface credibility* (μ_x_=4.43, σ_x_=1.12) was rated highly. Patients with CA did not want to see advertisements in an mHealth app. However, patients also expressed an understanding that the development and maintenance of such an app comes with a cost and that it had some form of payment:

That’s the stories of all apps, either you pay or you get ads. I would prefer to not have ads and pay for the app. I would think that the pharmaceutical and medical sector could also learn from the data. Everyone gets better.

Patient 3

*Verifiability* (μ_x_=3.50, σ_x_=1.48) and *Expertise* (μ_x_=3.23, σ_x_=1.43) received mixed scores. We polled the importance of listing the people who contributed to the app and or means to verify the content of the mHealth app. Some patients found this highly valuable, but others did not care much:

I would like to be able to validate the content of the app. You can read enough on the internet that isn’t true. I would like to know for sure that the content is scientifically grounded. […] I only know a few people in one hospital, I won’t recognize any one of the specialists listed in the app so I don’t think that’s useful.

Patient 3

I only know one rheumatologist in one hospital. If this application would be used nationwide, I wouldn’t recognize the people in the app anyhow.

Patient 3

*Styling* (μ_x_=2.97, σ_x_=1.43) equally received mixed scores; some patients liked being able to add a picture or change the background, whereas other patients were indifferent. Overall, it seemed like a nice feature to have but not an essential feature:

This is OK for me, but it is not necessary a 3.

Patient 18

#### Incentivization

**Figure 6:**
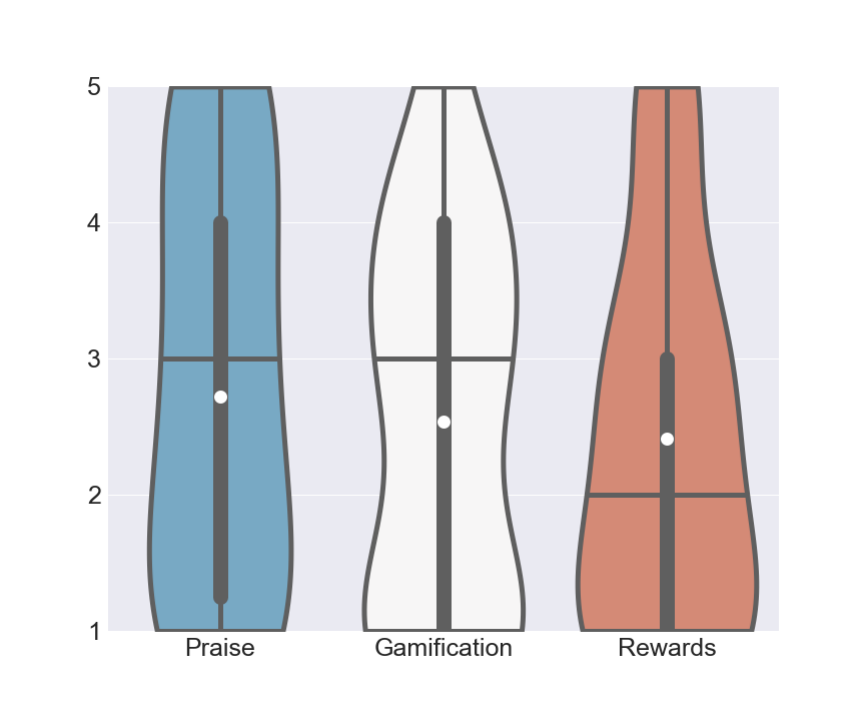
Scores of features in the group of Incentivization. The white dot denotes the mean value, the horizontal black bar denotes the median value, the thick black bar denotes the interquartile range and the thin black bar denotes the 95% confidence interval.

The theme of incentivization contains features aimed at increasing a patient’s motivation to execute a certain behavior (Figure 6) by providing rewards and giving praise by adding playful elements through gamification. Overall, this theme did not receive positive scores; in fact, none of the features’ mean score scored higher than 3.

*Praise* (μ_x_=2.83, σ_x_=1.49), *Rewards*(μ_x_=2.40, σ_x_=1.38), and *Gamification* (μ_x_=2.53, σ_x_=1.36) received mixed to negative evaluations. *Although s* ome patients liked a positive message offered via the app to motivate them, most patients categorized these as *extrinsic* motivators. They emphasized that they engaged in disease actions such as physical therapy to get better not to receive incentives:

Rewards are not necessary for me, I do my physical therapy because it’s good for my health.

Patient 18

Getting praising messages is less important than actually reminding me to sit straight or do my exercises. I would like it more when I get a message telling me I’m doing something wrong. [...] Those rewards are not going to take away my pain.

Patient 3

That’s for children. Although, it might be useful for some people... maybe children with CA.

Patient 27

#### Social Interaction

**Figure 7:**
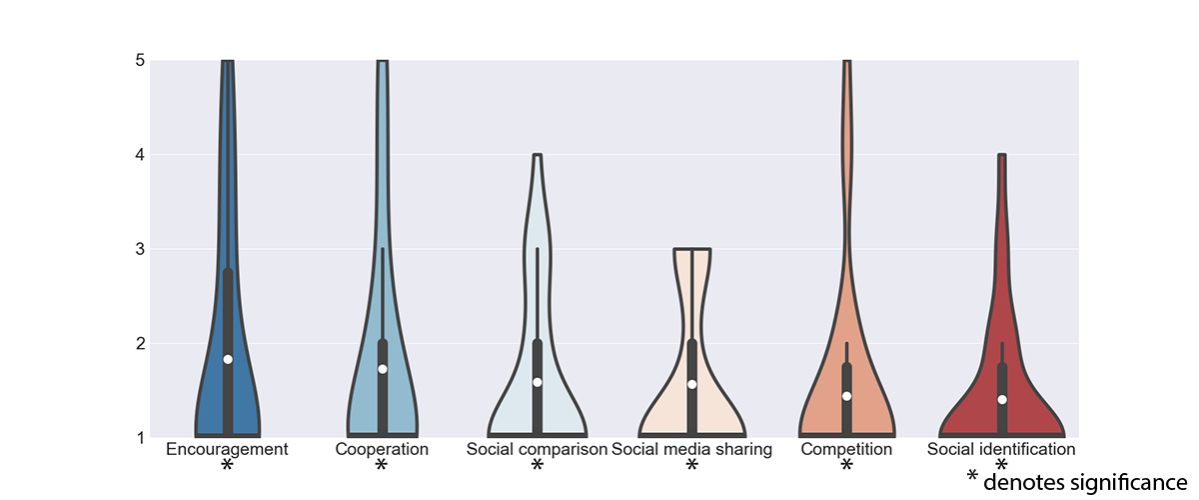
Scores of features in the group of Social interaction. The white dot denotes the mean value, the horizontal black bar denotes the median value, the thick black bar denotes the interquartile range and the thin black bar denotes the 95% confidence interval.

The group of social interaction contains features related to interacting, sharing, and comparing via social media or directly with others through the app (Figure 7). This theme scored the lowest of all themes, with all 6 features’ average score being below 2.

Receiving *Encouragements* from friends and relatives (μ_x_=1.90, σ_x_=1.30), *Cooperation* (μ_x_=1.80, σ_x_=1.22), and *Competition* (μ_x_=1.50, σ_x_=1.02) all received significant low scores. Patients felt their disease was of a personal matter, and certainly, patients did not want to be a burden to others:

I practice for myself, not to compare myself to others, like “Oh look I can’t do this, and this patient can.” What is important to me is what I can do.

Patient 15

When I’m exercising, I’m exercising for myself. I don’t need to involve others.

Patient 22

*Social media sharing* (μ_x_=1.53 σx=0.85), *Social identification* (μ_x_=1.40, σ_x_=0.76), and *Social comparison* (μ_x_=1.57, σ_x_=0.88) received a similar significant low score. While acknowledging that other patients may find this useful, patients with CA in our sample expressed a strong dislike, often explaining that they felt their disease was a private matter not to be shared via social media. They also did not think there was much to be learned from others as every patient experiences CA differently:

The people in my surroundings know my condition and want me to tell them as little as possible about my disease. I don’t want to bore them with my condition.

Patient 3

It’s possible that other people need to see this kind of information but I am not interested in seeing this type of data.

Patient 3

I guess this could be interesting [for others] but personally, this is a medical condition and is differently for everyone. I would not like to share it on the internet.

Patient 26

#### Core Concept: Every Patient Hurts in His or Her Own Way

To further our insights into the quantitative data, that is, scorings, we conducted an additional round of qualitative analysis. We identified that throughout the interviews, patients stressed that CA is a lifelong disease with periods of chronic pain and fatigue flaring up, often invisible yet profoundly impacting the patient and his/her surroundings. The way the disease unfolds over time and manifests itself in the patient and social environment is different for every patient, and patients with CA are well aware of this:

This disease is personal for everyone and so different to everyone.

Patient 20

Everyone has pain in his own way.

Patient 16

This may be useful if it is personalized. You have to be able to make changes or signal when it is going too fast. Because this is really different for every patient. A healthy person can build endurance but someone who has pain, like me − I feel better one day compared to another− has to be able to get a pass from time to time. So, a 3 if I can make changes and indicate why and flag where it hurts.

Patient 3

This awareness that no 2 patients with CA are alike also surfaced in the often careful articulation of scorings; our participants voiced their opinion clearly but then added that other patients with CA might have a different view:

I don’t need this. But it could be that there are patients that need this. It is, of course, different for everyone.

Patient 3

**Figure 8:**
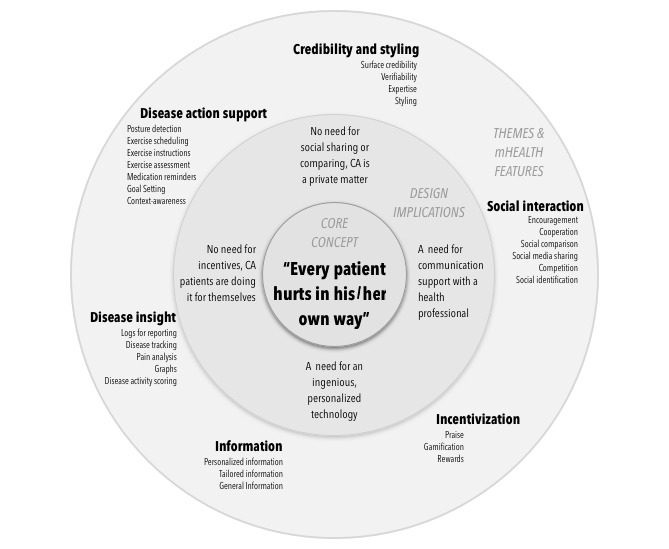
Image illustrating the relation of the core concept to design implications and themes and mHealth features.

Hence, *all patients hurt in their own way*. Understanding that each patient with CA has a unique experience of living with this chronic and painful disease, with frequent invisible manifestations of symptoms, allows for a deeper understanding of their evaluation of self-management features. On the basis of this understanding, we derived the following implications for the design of mHealth apps, illustrated in Figure 8.

#### Design Implications

##### No Need For Social Sharing or Comparing, Chronic Arthritis Is a Private Matter

As mentioned earlier, patients explained that their disease was a personal matter. Moreover, many patients struggled with the social acceptance of the disease and did not want to burden their friends, family, or other patients. Hence, patients with CA saw no use in sharing or comparing the disease status or reaching out for social support:

People know my disease and prefer me to mention it as little as possible. I do not want to be a drag.

Patient 3

The pain can be excruciating but what I want to emphasize is that CA patients have to face a second struggle, and that is the acceptance by their environment [...] It is hard to explain every time that “no, today I cannot join you for a walk, no I cannot participate in this fun activity.” This causes a psychological pressure. I have often been confronted with people saying “you are simply using the disease as an alibi to do nothing all day.”

Patient 28

That I would like to see [receiving encouragements]! When I was lying in the hospital for 9 months, even my own mother did not come for a visit.

Patient 9

I like to keep this to myself. My disease should not dominate my social encounters.

Patient 20

##### No Need For Incentives, Chronic Arthritis Patients Are Doing It For Themselves

Patients emphasized consistently they were engaging in disease management activities for themselves, to get better. Incentives in the form of rewards or praise would not help them get better and certainly not take away the pain. Consequently, they saw no use for *external* motivators coming from others or from an app:

I exercise for myself, not to compare myself to others, not “Oh, I can only do this, and that other patient still can do more.” The most important thing to me is what I can do myself.

Patient 15

To be reminded to maintain a certain posture is more important than receiving “Congrats!” [...]. I prefer to be reminded that I am doing something wrong, I do not need to be rewarded.

Patient 3

I don’t need that [rewards]. I’m doing this because it is good for my health, for my well−being.

Patient 18

##### A Need For Communication Support With a Health Professional

The highest rated feature in our study was *Logs for reporting*, a feature that supports communication with health professionals. Again, from an understanding that every patient with CA is different, it is important for them to make health professionals understand the specific peculiarities they are confronted with. Patients frequently reported forgetting disease-related events during the months between doctors’ appointments. Some patients addressed this issue by keeping a paper diary in which they noted events such as pain, stiffness, and medication intake. By being able to keep track of such events (logs and graphs), this may help specialists to provide a better diagnosis and hence the best possible care:

It seems a useful feature because you forget thing rapidly. When [the healthcare professionals] ask you questions but you have already forgotten that something has happened.

Patient 29

Yes, because the rheumatologist is better able to follow−up on your condition, and there is less chance of coming to a wrong conclusion.

Interviewer: Do you mean that the rheumatologist then has a better picture?

Patient 30

Yes, the doctor is better informed and will be more able to tailor the treatment.

Patient 30

##### A Need For Ingenious, Personalized Technology

Finally, many participants expressed reservations about the extent to which sensor and phone technology would be truly able to realize this *promise* of personalized self-management features. Patients were often skeptical and gave high scores in a *conditional* manner. Patients who ended up giving a lower score often did not believe that the app would be able to be technically performant or be personalized enough:

If the content is very heavily personalized to my needs, I would like this. But only if I can still adapt the schedule based on my progress because I might not feel well every day

Interviewer: Sensors measure whether you execute an exercise the right way

Patient 3

And how exactly would that work?Interviewer: Well, your mobile phone would connect to sensors to measure what movements you make. So the phone can do this? Wow, that would be great then, a 4.

Patient 21

It has to be quite ingenious to work, for now, I don’t know.Interviewer: Well, that’s the purpose.

Patient 3

Well it if works, I say OK, a 5, but I doubt it a bit. My physical therapist can tell me: “OK, you can bend till there, but if you feel you can’t, please stop.” If an app is supposed to give these instructions... Personally, I think that will be very hard to realize. There are so many different variants of arthritis that it will be really hard to program.

Patient 3

Patients with CA value this bond with physical therapists who could deliver highly personalized therapies. Many patients stressed that they valued the face-to-face contact and would not like to see the therapist be replaced by an app:

We have known each other for years, we do these exercises together. When we are lying on the ground and I doubt how to do it, then I look at how he is doing it. You don’t lose time because you need to practice that hour anyhow. Otherwise, you would need to look at an instructional video.

Patient 3

## Discussion

### Principal Findings

To summarize our findings, patients with CA valued the use of self-management features that support active and direct disease management. In this regard, they welcomed features that support medication intake, posture detection, and physical exercise. Patients also welcomed features that help them keep a close watch on their disease status and contribute to their health professional’s understanding of their disease. In this regard, they liked to have logs to communicate and analyze disease parameters such as pain and provide doctors with more insights on when and how this was manifested. The need for personalized and tailored insights was also reflected in the *information theme*. These findings are in line with the findings of earlier studies [15,22-24,33,34]. Interpreting these results in light of the self-management roles identified by Lorig et al [35], it becomes apparent that our sample of participants finds value in those mHealth features that support medical management tasks (ie, tasks to manage the condition such as taking medication, adhering to a special diet, or using an inhaler).

However, the patients with CA in our sample did not support features that support role management (ie, maintaining, changing, and creating new meaningful behaviors or life roles) or emotional management (learning to manage emotions such as anger, fear, frustration, and depression). In contrast with aforementioned studies, we did not find support for embedding features relating to social interaction [20]. This finding may be counterintuitive at first sight as it is generally acknowledged that social support is beneficial for a patient’s therapeutic trajectory [24,35-37] and paramount for a patient’s acceptance of the disease [38].

One possible explanation could be the difference in exposure to peer support and/or the use of social networking technology between younger and older age groups, as described by Vaterlaus et al [26]. However, by acknowledging that all patients with CA hurt in their own way, another possible cause may relate to disease duration: patients who have lived with CA for a longer time have learned to manage and understand the peculiarities of their disease as well as the impact on their social environment. Our sample of participants represents an older group of patients who have experienced *the waxing and waning* of the disease [35]. Although adolescents might be on their way of getting control over their condition, and actively looking for support from peers and or external confirmation through incentives, older patients with CA may have come to realize that their disease is a personal matter and may try to limit the *direct* impact on their social circle. It may be that for older patients with CA, acceptance is promoted, not by centralizing the disease itself but rather by undertaking meaningful activities in a social context despite the disease [39-44].

A third possible explanation may be that some of the studies that find positive evaluations of social interaction assume an in-person delivery, whereas the features we researched are inherently delivered through an mHealth app. Patients may prefer social interaction in real life but may not want the same interactions to occur through the means of an mHealth app.

In addition, our study did not find support for including mHealth features with regard to incentivization (ie, praise, rewards, and gamification). This is again in contrast with earlier studies [22-24]. Again, it may be that our older sample has come to live with *shifting perspectives* where sometimes wellness and sometimes illness move to the foreground [45]. It has been well documented that the major concern of patients with arthritis is pain management [46], and this was no different among our sample. As the disease progresses and patients age, pain management may become increasingly central to disease management of CA. Considering this, the use of *playful* extrinsic motivators may be considered irrelevant; our sample emphasized how such features would not help combat pain.

Finally, the different outcomes may also be the result of different research methods. Whereas prior studies rely on focus groups where participants explored and discussed possible features, our method started from an individual rating of given features. Often, our patients gave a negative scoring but added that other patients may feel differently. Interestingly, while acknowledging differences among patients with CA, patients in our sample were univocal in their dismissal of incentivization and social interaction features. These findings show the importance of inclusive methods in the development of mHealth apps. Although the BCT of Michie et al [47] and PSD model of Oinas-Kukkonen et al [9] offer inspiring models enlisting myriad BCTs or persuasive principles, an additional step is required to select those most preferred by specific audiences.

To conclude, this finding promotes a participatory design process for mHealth apps, involving patients, health care professionals, and developers in the creation of the app, but it equally promotes empirical measurement [48] and preference ranking of features [49]. Using a blend of inclusive methods ensures that those features deemed most useful by all stakeholders (caregivers and patients) end up in the app [50].

### Limitations and Future Work

We interviewed 31 patients and asked them to rate 28 features of an mHealth app for patients with CA. We described these features and gave extra information when asked for. Even though these steps were taken with care, some features may have remained elusive for patients to imagine. This may have been further influenced by a lack of past experience with mHealth and information and communication technology. It may have been difficult for some patients to see how these features would unfold and impact them. Unfortunately, we did not measure internet use, technology habits, and education or occupation and cannot verify how this impacted attitudes of patients with CA toward mHealth features. Hence, we suggest future studies to ask for this information. We also suggest future studies to provide examples of features being incorporated into existing apps or prototypes, for example, in the form of screenshots, to make them more tangible.

Furthermore, as we used the same order of self-management features for all patients (see Textbox 1), we may have introduced order effects. It may have been that patients with CA experienced fatigue toward the end of the interview and provided less information and/or different scorings. However, the richness in the results from the qualitative data analysis suggests that if there was an order bias, it was small. Moreover, owing to the semistructured nature of the interviews, whenever patients felt the need to digress from the order and express ideas differing from the fixed list of questions, they were free to do so. Our interviews show that they frequently did.

Finally, although we aimed for a heterogeneous sample of patients with regard to age, gender, and disease parameters, it is still a predominant white sample. With a median age of 53 years, living on average for 21 years with the disease already, the patients with CA in this study represent an older CA population. Hence, extrapolation to other younger adult populations needs to be done with care. In particular, ratings toward incentives, gamification, and social interaction may differ. On the basis of the psychological processes underlying the development of chronic pain (eg, fear avoidance versus fear endurance [46,51]), other researchers have shown the presence of subpopulations in chronic patients [43,52,53]. Further research could investigate how different subtypes further impact preferences for features and how mHealth apps may need further tailoring. Follow-up studies with different patient populations from different genders, ethnic backgrounds, socioeconomic status, and with different disease subtypes and/or comorbidities are needed.

### Conclusions

Data emerging from 31 interviews with patients with CA provided valuable insights into which mHealth app features are favored and which are disliked by patients. Patients had strong negative opinions regarding social features, stemming from the individual nature of managing one’s disease. They often remarked how everyone’s disease progress is different and how they did not want to bother others with their suffering. In addition, they did not want to receive incentives for completing disease-related actions as they claimed to be intrinsically motivated to get better. They did, however, have a strong preference for features, which enable them to keep better track of their condition and report these data to their health care professionals. They favored receiving tailored information, based on their own data, but at the same time questioned the possibility for an mHealth app to achieve this level of tailoring. We hope the results of this research can inform mHealth app developers as to which features are most valuable to include in an mHealth app for patients with CA.

## Abbreviations

BCT: Behavior Change Technique
CA: chronic arthritis
JIA: juvenile idiopathic arthritis
mHealth: mobile health
PSD: Persuasive System Design
